# The Role of Individual and Small-Area Social and Environmental Factors on Heat Vulnerability to Mortality Within and Outside of the Home in Boston, MA

**DOI:** 10.3390/cli8020029

**Published:** 2020-02-07

**Authors:** Augusta A. Williams, Joseph G. Allen, Paul J. Catalano, John D. Spengler

**Affiliations:** 1 Department of Environmental Health, Harvard T.H. Chan School of Public Health, Boston, MA 02115, USA; 2 Department of Biostatistics, Harvard T.H. Chan School of Public Health, Boston, MA 02115, USA; 3 Department of Data Sciences, Dana-Farber Cancer Institute, Boston, MA 02215, USA

**Keywords:** heat-related mortality, built environment, urban resilience, extreme heat, climate change, urban heat island

## Abstract

Climate change is resulting in heatwaves that are more frequent, severe, and longer lasting, which is projected to double-to-triple the heat-related mortality in Boston, MA if adequate climate change mitigation and adaptation strategies are not implemented. A case-only analysis was used to examine subject and small-area neighborhood characteristics that modified the association between hot days and mortality. Deaths of Boston, Massachusetts residents that occurred from 2000–2015 were analyzed in relation to the daily temperature and heat index during the warm season as part of the case-only analysis. The modification by small-area (census tract, CT) social, and environmental (natural and built) factors was assessed. At-home mortality on hot days was driven by both social and environmental factors, differentially across the City of Boston census tracts, with a greater proportion of low-to-no income individuals or those with limited English proficiency being more highly represented among those who died during the study period; but small-area built environment features, like street trees and enhanced energy efficiency, were able to reduce the relative odds of death within and outside the home. At temperatures below current local thresholds used for heat warnings and advisories, there was increased relative odds of death from substance abuse and assault-related altercations. Geographic weighted regression analyses were used to examine these relationships spatially within a subset of at-home deaths with high-resolution temperature and humidity data. This revealed spatially heterogeneous associations between at-home mortality and social and environmental vulnerability factors.

## 1. Introduction

Global climate change is increasing the frequency, duration, and severity of extreme heat events around the world [1,2]. Extreme heat is one of the most severe public health impacts of climate change, resulting in increased mortality in many geographic locations [3–6], including in Boston, Massachusetts (MA), USA, the focus of this study [7–11]. In Boston, MA, of the annual number of hot days above 90 °F is expected to increase from 11 (1971–2000) to 40 days by 2030, and potentially 90 days by 2070 [12]. Projections have shown that that heat-related mortality has the potential to triple in the Northeast United States [13] without adequate climate change mitigation and adaptation strategies being implemented, with the effects being disproportionally experienced by the most vulnerable individuals.

There has been significant research done to further the concept of heat vulnerability, to determine and understand which individuals and communities are most vulnerable to the impacts of extreme heat. Much of this literature has focused on social factors, such as income, race, sex, education, and age, to determine vulnerability. For example, reviews of heat and mortality epidemiologic studies have found that non-Hispanic black individuals, women, those of a lower socioeconomic status, those with preexisting medical conditions (e.g., diabetes), elderly individuals over 65, and infants and children under 5 have been found to be most vulnerable to poor health outcomes, including death, during extreme heat events [4–6,14–16].

One group of factors that determines much of our exposure to extreme heat are the natural and built environments. The urban heat island (UHI) effect occurs when land surface temperatures in an urban area are higher than the surrounding rural or suburban areas, and an urban design with enhanced urban canyons, greater use of materials of high thermal absorption (e.g., asphalt, brick), and densely built areas can create temperatures much higher than surrounding areas and prevent urban areas from cooling at night. Furthermore, buildings modify our exposure to ambient temperature conditions, either exacerbating or mitigating indoor heat exposures given the same ambient conditions [17].

While buildings have become more energy efficient, a higher thermal insulation and greater airtightness have increased the risk of potential overheating during periods of power outages [17]. These increases in energy efficiency in heat-dominated climates, like that of Boston, MA, may potentially be adversely impacting the resiliency of buildings to extreme heat during power outages [18]. Overheating is a great risk in times of power loss when mechanical cooling may not be available. The risk of major and widespread power outages that affect a large number of customers and that would result in “heat disasters” appears to be growing [19]. Research has shown that the summertime overheating of residential buildings may increase by upwards of 25% by the middle of the 21st century [20].

As this body of work has progressed, some attention has been paid to other factors in the surrounding urban environment that may exacerbate or mitigate the underlying social vulnerability to extreme heat. Some of the risk factors associated with building and urban design have been incorporated into previous heat vulnerability research. For example, living in a multi-family apartment building has been found to increase the incidence of poor health outcomes during extreme heat events [21,22]. Building materials with a high thermal mass prevent a building from passively cooling during the overnight hours, increasing the need for cooling during subsequent days [23,24]. Having urban tree canopies can keep surrounding buildings cool, reducing the electricity consumption from cooling demands usage, while also keeping communities cooler and mitigating heat stress-related health conditions [25]; additionally, surrounding greenspace can reduce vulnerability to heat morbidity and mortality [16].

Using air conditioning (AC) has been the primary defense to extreme heat exposure indoors. However, solely relying on AC is currently not an equitable or sustainable adaptation strategy. A recent study in New York City (NYC) found that the odds of not having AC were found to be greatest for non-Hispanic black people and those with low-to-no income [26], and only 50% of NYC public housing residents have access to AC [27]. Approximately two-thirds of Massachusetts residents lack central AC at home, while 20% of households lack any type of AC [28]. Furthermore, the electricity used to power AC produces health-harmful pollutants, which are increased on hot days from enhanced power production [29]. Lastly, the refrigerants found within AC systems contain hydrofluorocarbons, a greenhouse gas more potent than carbon dioxide.

Despite spending up to 90% of time within buildings and built environments that largely determine our exposure to ambient conditions, few heat vulnerability and adaptation assessments include widespread information on buildings and the built environment. Although a framework for assessing heat vulnerability has been created [15], the past application of this at the local level found that only about half of all census tracts were correctly characterized as being vulnerable [30]. This demonstrates the need for the tailoring and implementation of heat vulnerability assessments at the local level. Furthermore, many existing heat vulnerability assessment frameworks and associated studies have used a single ambient meteorological measurement to determine exposure for an entire city. Since buildings and the urban environment modify our exposure to extreme temperatures, the use of ambient temperatures to define heat exposure has resulted in temperature exposure misclassification as this does not adequately characterize the temperatures people are experiencing indoors.

In 2015, a Boston-based heat vulnerability and urban heat island assessment, prepared for by the Trust for Public Land, was conducted by students at Tufts University [31]. This plan outlined several solutions that would reduce Boston’s heat vulnerability, mainly through community engagement and adaptations to the built environment and was largely based on helpful and informative case studies of strategies implemented here and elsewhere in the US. While this assessment included some built environment factors like land use and central AC penetration, addition local research is needed on the role of buildings and the built environment in driving heat vulnerability in Boston in order to most effectively enact these interventions.

In this study, we aim to further local heat vulnerability research by incorporating neighborhood built and natural environmental characteristics in combination with social factors to assess vulnerability to mortality on hot days. A case-only analysis was used to examine whether the mortality on hot days in Boston, MA varied based on individual and small-area environmental and social characteristics at a variety of temperature exposure definitions. These relationships were then further explored spatially across Boston with a gridded climate dataset to enhance temperature exposure definitions. This comprehensive assessment of the underlying vulnerabilities and their social and environmental drivers will be vital when implementing heat action planning in the future.

## 2 Materials and Methods

Hourly meteorological data was accessed for the Boston Logan International Airport via the National Centers for Environmental Information. The daily maximum ambient temperatures (T_MAX_) ≥ 90 °F were used as a binary measure of it being a “hot day”, a common temperature threshold used to enact local cooling strategies in Boston. Recent evidence in Boston, MA has found that health impacts, including mortality, increase at daily maximum temperatures below 90 °F [32], so T_MAX_ ≥ 85 °F was used to define “warm days”. The heat index (HI)—a measure of the real feel temperature after the relative humidity is considered with the actual air temperature [33]—was also considered by using days where HI_MAX_ ≥ 86 °F, which was the 95th percentile of the HI_MAX_ during the warm season in Boston, from May to September.

These assessments use single day exposures, and are consistent with findings that the mortality risk or effect modification of heat-mortality relationships increase on individual days of extreme heat [5,34–36]. These temperature metrics are also at values that are below the current heat advisory (HI of 95–99 °F for at least 2 h over 2 consecutive days), or heat wave (at least 3 days where T_MAX_ ≥ 90 °F) criteria, which trigger local messaging and communications, as well as city-wide interventions (e.g., opening of cooling centers). All analyses were conducted using only the warm season (May to September) data, to remove any seasonal confounding that exists between the temperature and mortality.

Small-area neighborhood data was assigned at the census tract (CT) level for each at-home mortality record, corresponding to the 2010 Census. CTs are small, relatively permanent subdivisions, usually include around 4000 people, and are designed to be “homogeneous with respect to population characteristics, economic status, and living conditions” [37]. CT-based assessments of the socioeconomic status (SES) have been found to be adequate measurements for individual estimates in MA [38]. There are 178 CTs in the City of Boston, but 14 of these CTs have little or no population, leaving 164 CTs for analysis. We obtained data on the CT social characteristics including the population density, inclusion of utilities in rent, the Gini coefficient for income inequality, and unemployment from the 2010 Census [39]. The proportion of those in each CT with a disability, older adults ≥ 65 years, children ≤ 5 years, non-Caucasian (non-Hispanic whites), low income, limited English proficiency, and with medical illnesses was accessed from the 2016 City of Boston’s Climate Ready Boston Social Vulnerability Data, which draws upon the 2008–2012 American Community Survey 5-year Estimates [40].

We obtained data on the CT environmental characteristics, including the availability of street trees in 2011, from Boston Open Data [41], the 2005 impervious surface fraction and mean albedo from MassGIS, and as summarized by the Boston Area Research Initiative (BARI) [42]. 2017 building assessments from the City of Boston Assessing Department and as summarized by BARI provided CT-level summaries of residential buildings, including the decade/year built or last renovated. BARI also provides a mean residential energy efficiency score, which is an aggregated variable based on the age of the building, the heating system, and the cooling type, with higher values indicating a more energy efficient residence. We also used the mean value of the residential building/land and per area [43]. When evaluating the local real estate prices in Boston, triple decker homes and luxury apartment buildings, which are 2 common residential building archetypes in Boston, were found to have the highest value per area.

The data on all deaths occurring in Massachusetts for the period January 2000-December 2015 were obtained from The Commonwealth of Massachusetts Executive Office of Health and Human Services Department of Public Health. The data included the primary causes of death, classified using the International Classification of Disease, 10th Revision (ICD-10) codes of age, sex, race, place of death, education, occupation, and industry of work. All deaths, regardless of the primary causes, were included in this analysis given the wide range of health outcomes that can be negatively impacted or exacerbated by extreme heat [35,44–51]. All deaths from 2000–2015 that occurred at home or outside of the home (excluding those that occurred an inpatient or nursing facility) were included in this analysis. Deaths that occurred at home were used in assessing the at-home heat vulnerability based the surrounding neighborhood’s social and environmental characteristics.

A case-only analysis, which was originally proposed to examine gene-environment interactions [52], can also be used to study how slow-varying characteristics modify the effects of a time-varying environmental exposure on a specified outcome [53]. This methodology is applied here to analyze whether the mortality on hot days is modified by individual and small-area (CT) social and environmental characteristics throughout the entire 2000–2015 study period. Three levels of modifiers were assessed here, including personal factors, primary cause of death, and area-level characteristics, similar to Zanobetti et al. (2013) [54]. To conduct the case-only analyses, a logistic regression was used to examine whether modifiers of interest were associated with increased relative odds of death within or outside of the home on days where T_MAX_ ≥ 90 °F, T_MAX_ ≥ 85 °F, or HI_MAX_ ≥ 86 °F during the warm season. Individuals were considered to either have a personal modifier or not, or were dichotomized based on being either below the 25th percentile or above the 75th percentile of the values for neighborhood modifiers across Boston or dying from that specified primary cause of death, following previous studies using the same methodology [54]. Effect estimates and 95% confidence intervals are reported.

In addition to the temperature thresholds defined for the entire city using the airport weather station, we wanted to reduce a temperature exposure misclassification by assigning a local temperature estimate to each of the mortality records used in the geographically weighted regression. The Parameter-Elevation Regressions on Independent Slopes Model (PRISM) was used to provide higher spatial resolution (800 m^2^) modeled temperature values, derived from a combination of weather station data, elevation models, and other spatial datasets to generate gridded estimates of climatic parameters [55,56]. It relies on the assumption that elevation is one of the most important determinants of temperature and precipitation, but also factors in the horizontal distance between weather stations, the spatial closeness between weather stations, the vertical layer of the atmosphere, topography, proximity to a coast, and the ability of terrain to affect precipitation. The distribution of these factors in relation to Boston, MA, results in the coastal proximity and elevation being the 2 factors that are most influential to the local temperature and precipitation. More details on PRISM and its development can be found in Daly et al., 2002 and Daly et al., 2007 [55,57]. The PRISM dataset has been found to be suitable for epidemiological studies looking at extreme heat and health relationships [58,59]. The daily maximum temperature at an 800 m2 resolution along a uniform square grid was used to assign the maximum daily temperature for hot days where T_MAX_ ≥ 90 °F at Logan International Airport. This was also done for hot and humid days where HI_MAX_ ≥ 86 °F at the airport, using the daily maximum temperature and vapor pressure to assign an estimate of HI_MAX_ at the residence of each mortality record included in this subset of analyses. This daily data was available from 2003–2015, allowing us to capture all but 3 years of mortality data.

A subset of at-home deaths that occurred from 2003–2015 (i.e., those that had corresponding PRISM data) were spatially analyzed at the residential address using a geocoded latitude and longitude, and the outcome in the model was summarized as the daily tract-specific death rate per 100,000 people. The original objective of this research question was to pair building specific details with the death records, but too few death records with complete building information existed to do so, so area-level summaries were used for the building characteristics, along with the social and environmental parameters. A generalized linear model (GLM) with a Poisson distribution was used to fit the model to assess the impact of the temperature, and neighborhood social and environmental parameters on the tract- and day-specific mean at-home death rate on hot days. The linear model was fit using backwards stepwise fitting, first considering all covariates of interest in the model, eliminating those with *p* < 0.20. An ANOVA goodness of fit test determined that the nested model was an adequate fit compared to the full model. This same model was then used in the generalized geographically weighted regression (GWR) with a Poisson distribution with an appropriate covariance structure.

The use of mortality data was approved by both the Harvard TH Chan School of Public Health and The Commonwealth of Massachusetts Institutional Review Boards.

## 3 Results

During this 16-year study period, there was a total of 14,200 deaths in Boston that occurred at home. Death records missing a correct death date were excluded, and only records that occurred during the warm season (May–September) were kept in the analysis, leaving 6102 deaths that occurred at home. Excluding those deaths that happened within an inpatient or nursing facility, 34,404 deaths that had complete information during this time period occurred outside of the home (Table 1). Of the deaths that happened at home, 197 (8.5%) occurred on days with T_MAX_ ≥ 90 °F, 186 (8.0%) on days with HI_MAX_ ≥ 86 °F, and 475 (20.4%) on days with T_MAX_ ≥ 85 °F. The descriptive statistics of the CTs in Boston are provided in Table 1. A map of Boston neighborhoods is available in Figure S1, and maps of the distribution of each small-area social and environmental factor are provided in Figure S2.

When evaluating the PRISM data of the spatial distribution of daily high temperatures, there was an average 2.97 °F temperature difference (∆T_MAX_) throughout the City of Boston on days when the airport temperature data indicated that daily T_MAX_ ≥ 90 °F. The daily modeled vapor pressure and T_MAX_ were used to calculate the daily heat index on days when HI_MAX_ ≥ 86 °F at Logan International Airport. On average, the ∆HI_MAX_ was 8.1 °F across Boston, with the lowest HI_MAX_ and highest HI_MAX_ across Boston being 83.6 °F and 91.8 °F, respectively.

### 3.1. Case-Only Analysis

#### 3.1.1. Deaths at Home

There were not significantly higher relative odds of dying at home than outside of the home for all temperature exposure definitions: T_MAX_ ≥ 90 °F odds ratio (OR) = 1.01 (95% confidence interval [CI]: 0.91, 1.11), HI_MAX_ ≥ 86 °F OR = 1.06 (95% CI: 0.95, 1.17), and T_MAX_ ≥ 85 °F OR = 1.07 (95% CI: 1.00, 1.14). Compared with at-home deaths occurring on other warm season days, individuals living in CTs with a higher proportion of low-to-no income persons (OR = 1.30, 95% CI: 1.06, 1.59) or with limited English proficiency (OR = 1.29, 95% CI: 1.05, 1.57) had higher relative odds of dying at home on days with T_MAX_ ≥ 90 °F than those in communities without these traits. Individuals who died at home living in CTs with a higher assessed building value per area were more highly represented among all at-home deaths than on days with T_MAX_ ≥ 90 °F with an OR = 1.26 (95% CI: 1.04, 1.53) (Table 2).

When considering days where HI_MAX_ ≥ 86 °F, none of the personal or neighborhood social factors were significant modifiers between days where HI_MAX_ ≥ 86 °F and at-home mortality. However, individuals living in CTs with newer or more recently renovated residential buildings or a greater density of street trees in the CT had a lower relative risk of dying at home with OR = 0.77 (95% CI: 0.62, 0.95) and OR = 0.76 (95% CI: 0.60, 0.97), respectively (Table 2). There were no primary causes of death that were significant modifiers of this association on hot and humid days. There were also no significant individual, community social, or community environmental modifiers on days where T_MAX_ ≥ 85 °F for at-home deaths, but those whose primary cause of death was infection-related were more highly represented with OR = 1.69 (95% CI: 1.02, 2.80)

#### 3.1.2. Deaths Outside of the Home

When considering deaths that occurred outside of the home but not within an inpatient or nursing facility, we first examined all deaths that occurred within the city of Boston (including those that did not reside in the city of Boston).. On warm days, where T_MAX_ ≥ 85 °F, we saw that deaths from circulatory/heart-related disease were less represented in deaths outside of the home, with OR = 0.94 (95% CI: 0.89, 0.99). On these warm days, individual characteristics significantly modified the association between heat and deaths such that individuals over the age of 82 (the 75th percentile of ages represented in deaths occurring in Boston outside of the home) (OR = 0.93; 95% CI: 0.87, 0.98) had lower relative odds of death, but individuals younger than 52 (the 25th percentile) had higher relative odds of death (OR = 1.09; 95% CI: 1.02, 1.16). There was no significant effect modification for any factors on hot or hot and humid days (Table 3).

We then restricted these deaths outside of the home to just those that involved Boston residents. On hot days, where T_MAX_ ≥ 90 °F, deaths from substance abuse (OR = 2.88; 95% CI: 1.42, 5.86) and unknown causes (OR = 2.38; 95% CI: 1.13, 5.02) were more highly represented, as were those in CTs with a greater proportion of elderly individuals or with a greater CT energy efficiency score. On days were HI_MAX_ ≥ 86 °F, individuals in the youngest quartile of mortality records, below age 58, had greater relative odds of death, with OR = 1.53 (95% CI: 1.07, 2.18). On warm days, where T_MAX_ ≥ 85 °F, individuals under 58 were still more highly represented (OR = 1.31; 95% CI: 1.05, 1.62), and deaths from assault-related altercations had 1.79 times the relative odds of occurring on warm days (95% CI: 1.24, 2.58) (Table 4).

### 3.2. Geographic Weighted Regression

Geographic weighted regression analyses were used to evaluate the role of environmental and social factors in driving the spatial distribution of at-home mortality from 2003–2015 on hot days (n = 389) with the gridded climate data defining the local T_MAX_ at the residence of each death record. The nested GLM that was found to be an appropriate fit using the model-building criteria previously described was compared to the full model using an ANOVA goodness of fit test, as shown in Equation (1).


(1) $$\begin{array}{l} Total\;Daily\;Death\;Rate∼\beta_{0}+\beta_{1}\left(Albedo\right)+\beta_{2}\left(\frac{Street\;Trees}{CTArea}\right)+\beta_{3}\left(\frac{Value}{Area}\right)+\\ \;\beta_{4}\left(Impervious\;Surface\;Fraction\right)+\beta_{5}\left(Population\;Density\right)+\\ \;\beta_{6}\left(Prop.\;Disability\right)+\beta_{7}\left(Prop.\;Older\right)+\beta_{8}\left(Prop.\;Low\;Income\right)+\\ \;\beta_{9}\left(Prop.\;People\;of\;Color\right)+\beta_{10}\left(T_{MAX-LOCAL}\right), \end{array}$$

The covariates from the GLM showed that the density of street trees in a CT and the proportion of those in the CT who were older adults were significant predictors of the daily at-home death rate (Table S1). Applying the same GLM model to the GWR, the GWR model (Equation (2)) was found to have a pseudo R^2^ = 0.89.


(2) $$\begin{array}{l} Total\;Daily\;Death\;Rate∼\beta_{0}+\beta_{1}\left(Albedo\right)+\beta_{2}\left(\frac{Street\;Trees}{CT\;Area}\right)+\beta_{3}\left(\frac{Building\;Value}{Building\;Area}\right)+\\ \;\beta_{4}\left(Impervious\;Surface\;Fraction\right)+\beta_{5}\left(Population\;Density\right)+\\ \;\beta_{6}\left(Prop.\;Disability\right)+\beta_{7}\left(Prop.\;Older\right)+\beta_{8}\left(Prop.\;Low\;Income\right)+\\ \;\beta_{9}\left(Prop.\;People\;of\;Color\right)+\beta_{10}\left(T_{MAX_{local}}\right)+off\;set\left(\log\left(Population\right)\right), \end{array}$$

There were wide ranges in the value of the coefficients when examining them spatially, indicating relationships with at-home mortality on hot days that vary based on the local context. The mean CT albedo, population density, proportion of those with a disability, and the proportion of those with low-to-no income or who are non-Caucasian had local regression coefficients that were negative, indicating a lower daily mean death rate (Table S2). The median values of the local coefficients for the daily T_MAX_, trees/CT area, impervious surface fraction, and the proportion of those who are older adults aged 65 or older were positive, indicating a higher daily mean death rate.

**Figure 1.**
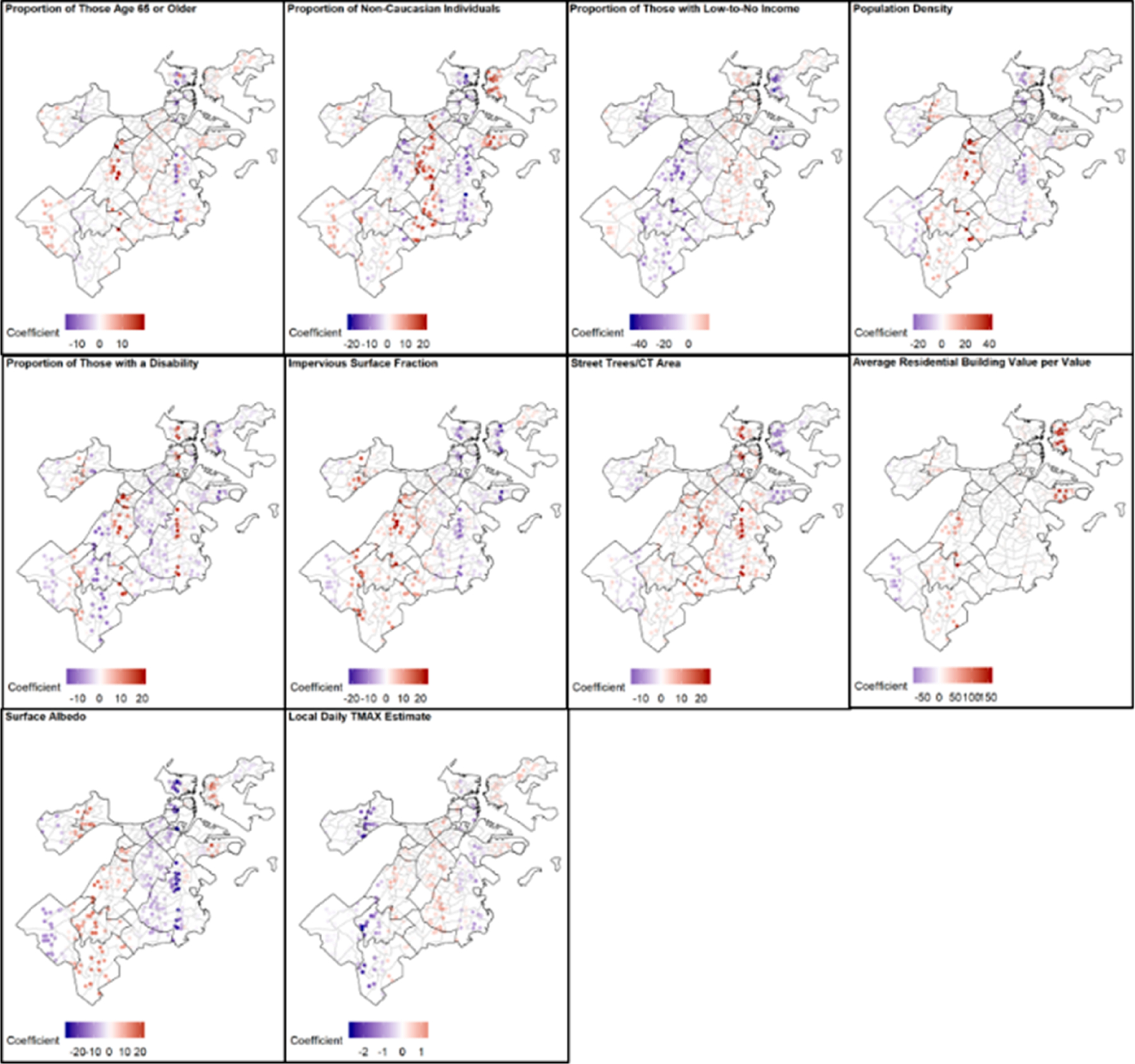
The local GWR coefficients, with blue colors representing negative coefficients (lower daily at-home mortality rate) and red colors representing positive coefficients (higher daily at-home mortality rate), in relation to daily at-home death rates on days where T_MAX_ ≥ 90 °F at Logan International Airport.

Figure 1 provides a map of the local coefficients for each of the covariates, included in the GWR model, that was restricted to just the deaths occurring at home on hot days. The strongest positive local coefficients (which would contribute to a higher at-home mortality) were seen with the proportion of non-Caucasian individuals in East Boston, Roxbury, and Dorchester, the population density in parts of Mission Hill and into Jamaica Plain, the proportion of trees per the CT area in Dorchester, the value of building per area in East Boston, and the surface albedo from Roslindale through Hyde Park. The strongest negative local coefficients were seen with the proportion of those with low-to-no income from Mission Hill south through Roslindale and Hyde Park, and the surface albedo from Charlestown through Downtown to Dorchester.

The same GWR analyses were used to evaluate the role of the environmental and social factors in driving the spatial distribution of the at-home mortality on hot and humid days, where HI_MAX_ ≥ 86 °F at Logan International Airport (n=358) with the gridded climate data defining the HI_MAX_ at the residence of each death record. A nested GLM that included the proportion of non-Caucasian individuals but excluded the population density and building value/area was found to be an appropriate fit using the model-building criteria previously described, compared to the full model using an ANOVA goodness of fit test, and is found in Equation (3).


(3) $$\begin{array}{l} \;Total\;Daily\;Death\;Rate∼\beta_{0}+\beta_{1}\left(Albedo\right)+\beta_{2}\left(\frac{Street\;Trees}{CT\;Area}\right)+\\ \beta_{3}\left(Impervious\;Surface\;Fraction\right)+\beta_{4}\left(Prop.\;Disability\right)+\beta_{5}\left(PProp.\;Older\right)+\\ \;\beta_{6}\left(Prop.\;Low\;Income\right)+\beta_{7}\left(Prop.\;People\;of\;Color\right)+\beta_{8}\left(HI_{MAX-LOCAL}\right), \end{array}$$

The covariates from the GLM for the days when HI_MAX_ ≥ 86 ° F showed that the impervious surface fraction and the proportion of elderly individuals were significantly associated with the daily at-home death rate (Table S3). Applying the same GLM model to the GWR, the GWR model (Equation (4)) was found to have a pseudo R^2^ = 0.75.


(4) $$\begin{array}{l} Total\;Daily\;Death\;Rate∼\beta_{0}+\beta_{1}\left(Albedo\right)+\beta_{2}\left(\frac{Street\;Trees}{CT\;Area}\right)+\\ \;\beta_{3}\left(Impervious\;Surface\;Fraction\right)+\beta_{4}\left(Prop.\;Disability\right)+\\ \;\beta_{5}\left(Prop.\;Older\right)+\beta_{6}\left(Prop.\;Low\;Income\right)+\\ \;\beta_{7}\left(Prop.\;People\;of\;Color\right)+\beta_{8}\left(T_{MAX_{local}}\right)+\\ \;\beta_{9}\left(off\;set\left(\log\left(Population\right)\right)\right) \end{array}$$

**Figure 2.**
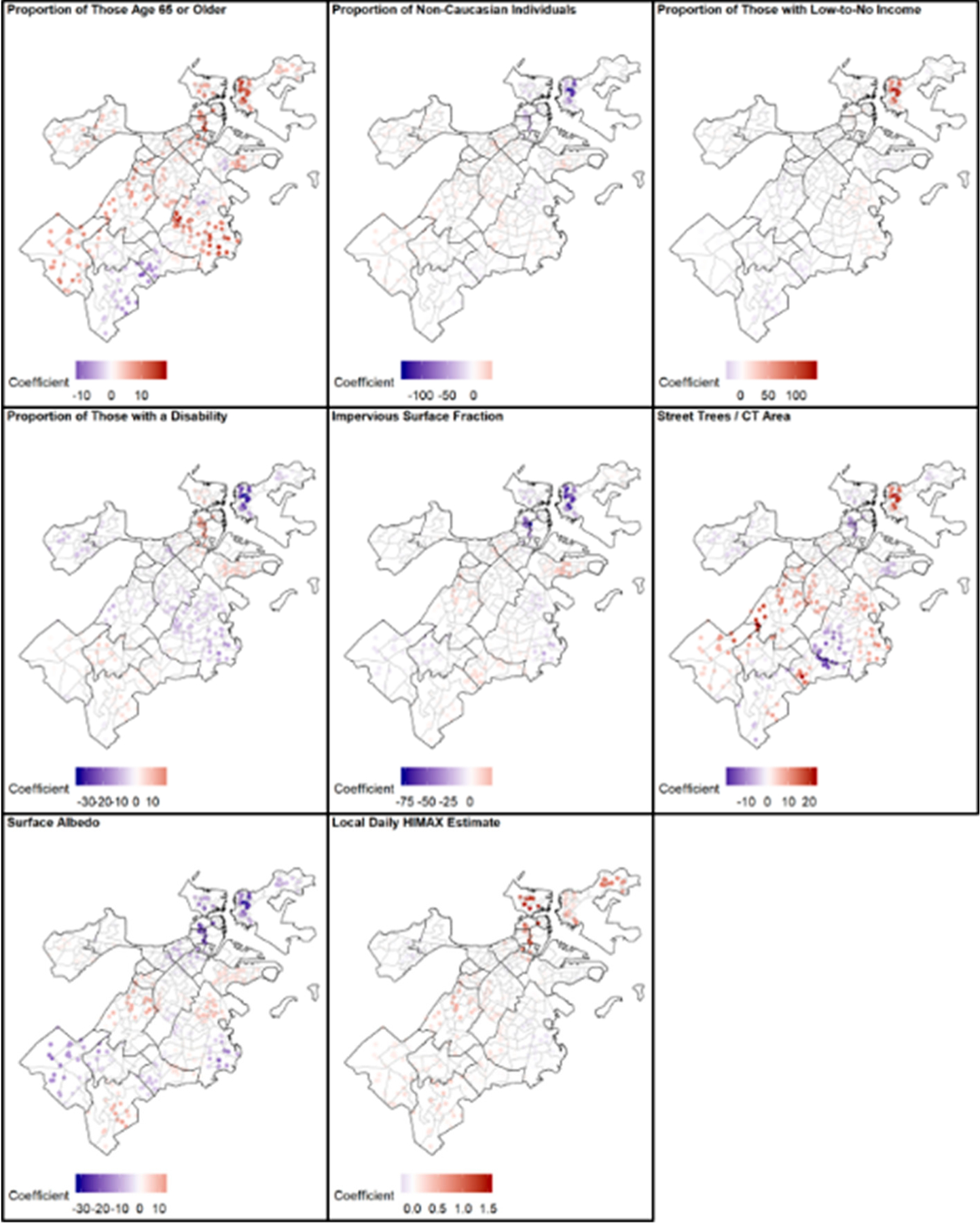
The local GWR coe_cients, with blue colors representing negative coeffcients and red colors representing positive coeffcients, in relation to the daily at-home death rate on days where HI_MAX_ ≥ 86 °F at Logan International Airport. CT denotes census tract.

Again, there is a spatial heterogeneity in the value of the coefficients (Table S4). Similar to those days when T_MAX_ ≥ 90 °F at the airport, the mean CT albedo, proportion of those with a disability, and the proportion of those with low-to-no income or who are non-Caucasian had local regression coefficients that were negative, indicating a negative association with the daily mean death rate on days when HI_MAX_ ≥ 86 °F at the airport. The median values of all the other local coefficients, including the local HI_MAX_, were positive, indicating a higher daily mean death rate. Figure 2 provides a map of the local coefficients for each of the covariates included in the GWR model that was restricted to just the deaths occurring at home on hot and humid days. Similar trends were seen in these analyses, with many of the strongest positive local coefficients seen with social and environmental vulnerability factors in East Boston and Dorchester.

## 4 Discussion

Heat vulnerability assessments have been found to provide valuable evidence for the design and strategic implementation of adaptation solutions that most effectively protect vulnerable populations. The results from this study provide valuable local heat vulnerability knowledge to Boston, MA, while also furthering the evidence of heat-mortality relationships in the Northeast US. This study examined several small area-level and individual social and environmental factors that are associated with an increased likelihood of dying at home during extreme heat events. In the case-only analyses, those living in CTs with a greater prevalence of well-known social vulnerability factors, like those with low-to-no income or with a limited English proficiency had increased relative odds of dying at home on a hot day during the warm season in Boston, MA. This supports much of the existing research on heat vulnerability [15,26]. However, some small-area environmental factors, like density of street trees and the more recently built/renovated residential buildings, were less represented among at-home deaths on hot days, which can inform adaptation strategies throughout the city.

There was not a significantly higher relative risk for elderly individuals or CTs with a greater proportion of elderly individuals, dying at home at any temperature definition, which is similar to past research on heat vulnerability in NYC [26], despite older adults’ enhanced susceptibility to extreme heat and previous findings documenting an increased vulnerability [4,5]. We hypothesize that these individuals, who are extremely vulnerable to heat-related mortality, may be more frequently dying within inpatient or nursing facilities, and were therefore not captured in this analysis. Furthermore, those who are oldest may be themselves aware of their enhanced susceptibility to heat-related medical complications, so may take precautions on these days (or have a support system that aids them in doing so).

Some environmental parameters, like more recently renovated/built residential buildings or a greater density of street trees, were able to reduce the relative odds of death for those dying at home on hot and humid days. However, other environmental factors, like the value of the residential building per its area (at-home deaths) and the CT residential energy efficiency (deaths of Boston residents outside of the home), were associated with higher relative odds of death. In Boston, an evaluation of local real estate prices revealed that triple decker homes had one of the highest values per area. These homes were historically used to house immigrant workers, and when their popularity surged in the 1980s it was common for them to be bought by absentee landlords and be poorly maintained [60]. Furthermore, apartment buildings are more energy efficient than single-family detached homes, and living in multi-family homes has previously found to be a risk factor for heat-related mortality in past severe heat events [21]. The specific context of an individual building should be taken into consideration when designing or retrofitting a building to better elucidate these relationships.

The small-area social and environmental factors surrounding the homes of those who died were not as important for deaths that occurred outside of the home as compared to those that happened at home. This makes sense, as the home neighborhood characteristics will likely not play as much of a role for deaths that occur elsewhere as they would for deaths that occur at home. However, Boston residents who were in the youngest quartile of deaths outside of the home were more highly represented within those that died on warm, as well as on hot and humid days. Future studies could investigate the breakdown of this lowest quartile, determining the relative risk of death for vulnerable populations that are young, such as children or occupational populations.

Interestingly, the temperature thresholds that were used did yield modifications by different primary causes of death. Deaths at home were more highly represented by those who died of heart disease or infection, which is similar to previous findings [4,61]. On hot and humid days, deaths from digestive-related issues were more highly represented among Boston residents who died outside of the home, which is also supported by the past literature [51]. Boston residents who died outside of the home during this time period had 2.88 and 2.38 times the relative odds of dying from substance abuse or unknown causes when T_MAX_ ≥ 90 °F days and 1.79 times the relative odds of dying from an assault-related altercation on days when T_MAX_ ≥ 85°F. Given that past research has shown that the heat-related mortality is frequently misclassified, and that sometimes proper causes are not listed at all, we believe that the high relative odds of dying from unknown causes presents a clear signal of heat-related mortality effects. Furthermore, research has demonstrated that aggressive and violent behaviors increase on hot days [62–65]. In Boston, there are significant increases in police, fire, and medical services across the city at temperatures of around 83–85 °F [66], which provides evidence that societal services are impacted by heat at these lower temperatures [67]. Our findings of increased relative odds of dying from substance abuse follow patterns seen in past studies in other geographic locations [68,69]. To our knowledge, these causes of deaths are not captured in local assessments of the public health impacts of extreme heat and mortality risk factors, despite some anecdotal information provided to us on these associations at the start of this study (personal communications with the City of Boston Public Health Commission).

The relationships between social and environmental modifiers of at-home mortality during extreme heat events varied spatially. When comparing this information to previous heat vulnerability assessments in Boston, MA, a few interesting trends emerge. A 2015 assessment of a heat vulnerability index found that 11 CTs in Boston had the greatest heat vulnerability despite access to cooling resources (e.g., cool centers, spray pads, etc.), including in East Boston, Chinatown, Fenway, the South End, Mattapan, Roslindale, and 5 CTs in Roxbury, all primarily driven by social and environmental vulnerability [31]. The area of East Boston that was found to be highly vulnerable also corresponded to areas of East Boston that were found to have an increased social and environmental vulnerability to extreme heat. These analyses did not find that Chinatown, Fenway, or the South End were particularly vulnerable to dying at home on a hot day. However, mortality is the most extreme outcome that is possible with extreme heat exposure, with deaths at home comprising a small fraction of all deaths, so these other neighborhoods may still be vulnerable to other negative health outcomes, if not to at-home mortality. Alternatively, with Boston’s recent adaptation of heat mitigation strategies, it would be worth investigating if any social or environmental interventions had taken place during this study period that could attenuate the at-home death rate on hot days in certain neighborhoods.

The 2016 Climate Ready Boston report highlights the CTs where the greater proportion of socially vulnerable individuals live in the city, including those who are non-Caucasian, of older age, younger age, with a disability or a medical illness, with limited English proficiency, or of low-to-no income. The neighborhoods of Dorchester, Roxbury, East Boston, Brighton, Downtown, South End, and Mission Hill appear to be the most vulnerable from these factors. While these neighborhoods have some overlap with the findings of this study—especially Dorchester, Roxbury, and East Boston—the others do not have as much overlap. Again, the results of this study are not saying that residents of these other neighborhoods are not vulnerable, but instead that they are not as socially or environmentally vulnerable as those Dorchester, Roxbury, and East Boston to dying at home on hot days.

The results of this analysis highlight the spatial variability of heat vulnerability factors, which would have been masked by traditional regression analyses that assumed spatially consistent relationships between all of these factors and at-home mortality. From this, interesting trends in some of the environmental vulnerability factors emerged, where in some areas features considered to be beneficial, like street trees, demonstrate a negative relationship with daily at-home mortality. We hypothesize that this may be related to the differential quality of environmental features that are present. One example of this is present with a discussion of the quality over quantity of street trees. In many neighborhoods, the GWR analyses suggest a positive association between the density of street trees and at-home mortality on hot days, insofar as the density of street trees was associated with a higher at-home mortality rate, which is counterintuitive to what one may expect. The quality of these trees is important in determining how much shading effect may be available, but we did not have quantitative information on the quality of the trees in each neighborhood. Figure S3 demonstrates some of the features of trees that may be influencing their role in shading residential buildings, including if they are in an ill state of health, if they are young trees, or if they are not equally distributed within neighborhoods (i.e., only located on one side of a street), all of which would limit the shading potential the trees had to offer. We are eager to explore the role of this building archetype and design and this street tree quality on indoor temperature exposures in future studies.

One of the most unique aspects of this research is the application of a gridded climate dataset to refine the temperature exposure throughout Boston. Many of the strongest coefficients, in either direction, align with Interstate-93 in the easternmost part of Boston. This boundary may also be indicative of the extent of the sea breeze on hot days. On these hot days, the mean wind direction at Logan International Airport during the day was 89.5°, indicating easterly winds off the Atlantic Ocean. Past analyses of Boston’s sea breeze have found that it is one of the most developed in mid-latitudes with peaks in July and August. Within the city of Boston, there is both a smaller bay breeze effect, as well as a larger sea breeze effect, which often does not penetrate much further inland than the coastline due to abrupt topographic shifts in western Boston [70]. We hypothesize that this boundary, seen stretching north-to-south from Downtown to Dorchester, may be due to the proximity to a major roadway, which has documented negative health effects on surrounding residents [71], or to stark changes in temperature resulting from a microclimatic effect, like a bay breeze or the marine layer, or perhaps to a combination of these elements and requires further research. By using a refined exposure assessment and capturing temperature variations across the city, we can distinguish this local climate effect, which influences heat exposure and thus heat vulnerability.

The results of this study highlight the differential social and environmental drivers of heat vulnerability in Boston, MA. The goal of this study was to demonstrate how our surrounding built and natural environment exists concurrently with our social environment and how it can provide an important point of intervention, relying less on changing behaviors, that can be used in tandem with the existing communication of heat-harm-reduction strategies. Locally, there have been active efforts to mitigate poor health outcomes during extreme heat events for those who have been found to be socially vulnerable. However, it is important to remember that the natural and built environment play a large role in determining an individual’s exposure to extreme heat and interact with the social factors to create unique vulnerabilities for each neighborhood. As was seen in East Boston, the proportion of street trees/CT area increased, had negative location coefficients in relationship to at-home mortality rate. A recent study found that nearly 80% of those living in almost 100 different US cities were in neighborhoods with less than 20% tree cover [25]. Most CTs in Boston are also below this percentage of tree cover [31]. Researchers then simulated in these cities an improved urban tree canopy and found that 245–346 deaths per year could be avoided, while also reducing building-related heat stress so that the cooling demand and attendant electricity consumption could decrease [25]. Lessons learned from the effective deployment of street trees in neighborhoods where trees have been shown to be protective of poor health outcomes may inform future adaptation strategies that increase the urban tree canopy most effectively.

Adaptation strategies like improved urban materials, cool roofs, evaporative roofs, and the increased density of street trees have the potential to reduce the range of summertime temperatures in the Northeast US and reduce the impact of the increased number of hot days that we are expected to experience by the end of the century [50]. The median age of residential buildings in Boston, MA is 54 years, and some of the most common residential building archetypes in Boston were predominantly built in the late 19th and early 20th centuries. Older buildings, which tend to use more traditional materials, have been found to have higher thermal masses [72], making them prone to overheating during times of power outages. Low-income individuals are more likely to live in homes that are overcrowded and/or that are of poor environmental and physical quality [73]. While these relationships between housing and public health are not new, demonstrating the quantitative effect that these built environment factors have on a climate change-relevant public health outcome provides local actionable evidence.

Currently, Boston has primarily relied on mechanical cooling to mitigate hot indoor temperatures, but without that cooling many of our residential buildings are not passively habitable and cannot provide safe and survivable indoor conditions without mechanical cooling in times of extreme heat [24]. In order to be the most resilient to the future extreme heat events of the next century, our residential buildings and surrounding built environments need to consider climate adaptive architecture and design that reduce the thermal loads of our urban areas. Planning for the health impacts of extreme heat in the design and retrofitting of buildings is vital for protecting public health in a changing climate. This will be important in urban areas, but also in those suburban areas and satellite cities around major urban centers that have been found to show a rapidly increasing vulnerability to extreme heat [74].

It is important to consider the limitations of these analyses. A case-only analysis is limited in that it only assesses the relative risk of mortality with individual and neighborhood characteristics, and not the greatest absolute risk. Additionally, in a case-only analysis you can only evaluate one modifier at a time, and we have evaluated many personal, social, and environmental factors. Given the large number of factors evaluated, there could be increases in a type I error that could potentially lead to the spurious identification of factors that modify the association between extreme heat and mortality, and we are unable to assess various combinations of environmental and social vulnerability. Although we have removed the effect of season, residual confounding may still be present if there is an interaction between season and any of the effect modifiers being examined. This study relies heavily on CT-level boundaries and information. There were likely changes in the social/environmental parameters over this study period, but only cross-sectional estimates that were available at different points in this study period were utilized to assign these parameters across an entire 15-year time period. Finally, one well known heat-mitigating environmental factor—the proximity to greenspace—was not included in this study, as Boston is only one of 2 cities in the US where 100% of the population is within 10 min from a park [75], so there was no significant variation amongst those deaths included in this study in relation to this environmental factor.

Furthermore, there are many ways to measure temperature and assess extreme heat. The temperature thresholds used within this study are supported by recent research, including findings by Guo et al. (2017), according to which daily maximum temperatures for defining a heat wave are better at predicting mortality than the minimum temperatures are, [76] as well as findings by Kingsley et al. (2016), which state that there are significant increases in mortality at temperatures from 75–85 °F in Rhode Island [32]. The thresholds used in these analyses also align with local heat interventions that are enacted in Boston during extreme heat events, including the opening of cooling centers and transportation for vulnerable persons, and they are therefore important to local heat responses. The use of modeled daily temperatures at the location of each of the residences examined in this study further reduces the temperature misclassification that is commonly present in heat-health research. However, we also acknowledge that metrics like the duration of extreme heat and whether or not an extreme heat event is the first of a season or not are important to consider [34], and that buildings influence heat exposure; consequently, further work is needed to fully remove any bias from temperature exposure misclassification.

Despite these limitations, some of which also exist in much of the preexisting heat vulnerability research, there are many strengths to the analyses within this paper. A case-only analysis that assesses the change in the heat-related mortality risk by individual and neighborhood characteristics can be used to create composite heat-related vulnerability indices to prioritize the most vulnerable neighborhoods based on social and environmental factors. This information can be used to compare how non-time varying characteristics—or characteristics that change very slowly over time—modify the effect of a time-varying environmental exposure like extreme heat on excess mortality. A case-only analysis that is focused on the warm season only also removes the effect of season, which reduces potential seasonal confounding, reduces potential confounding by variables typically associated with mortality (e.g., smoking), simplifies modeling, and reduces the model’s sensitivity to misspecification bias. By including some health-promotive covariates, like street trees and albedo, we can assess protective neighborhood features instead of just assessing harmful features. The inclusion of both positive and adverse covariates allows us to both focus on strategies that create more health-protective environments and to reduce harmful environments, respectively, for all. This study also took a vast body of heat vulnerability research and metrics that have been demonstrated to be important for heat-mortality relationships, and assessed them at a local level, where the context and scale that were considered were tailored to Boston, MA. Finally, the use of the gridded climate dataset allows us to reduce temperature exposure misclassification and better characterize local temperature variability throughout Boston.

Future studies on the specific local building archetypes and how these modify indoor temperature exposures will fill a gap that remains from this study and will provide the most actionable evidence when constructing or retrofitting buildings to be more heat resilient. As Boston is currently exploring altering building codes to enhance resilience to flooding and sea-level rising, it will be critical to evaluate these policy measures for extreme heat as well. Integrating this work with a simulated adaptation strategy implementation can elucidate which building-focused adaptation strategies will be the most effective at reducing poor health outcomes at home during extreme heat events.

## 5 Conclusions

Future climate change adaptation will be implemented at the local level, so decision makers need data and evidence at that scale to best inform policy and infrastructure decisions. This study examined mortality at home and outside of the home on hot days in Boston, MA. We evaluated how individual and small-area social, built environment, and natural environment characteristics modify the association between heat and mortality at three temperature thresholds. While some neighborhoods had a greater social vulnerability, some of the environmental factors examined were able to reduce the relative odds of death within and outside the home. While many neighborhoods have been found to be socially vulnerable in past studies, there are some neighborhoods—like Roxbury, Dorchester, and East Boston—where both social and environmental vulnerability exists, highlighting priority locations for the implementation of a wide range of adaptation solutions. Furthermore, even at temperatures below current local thresholds used for warnings, advisories, and local interventions, there were significantly higher relative odds of death from unknown causes, substance abuse, injury/accidents, and assault, most of which are not currently being incorporated into heat vulnerability assessments and adaptation planning.

## Supplementary Material

Supplemental Infor

## Figures and Tables

**Table 1.** Descriptive statistics for the Boston, MA warm season climate, analyzed deaths from all causes from 2000–2015, and small-area social and environmental parameters at the census tract (CT) level. The frequency (n) represents the number of days meeting that meteorological threshold or the number of deaths that met that characteristic.

Characteristic	
Warm Season Maximum Temperature [°F] (mean (range))	75.7 (46–103)
Warm Season Mean Temperature [°F] (mean (range))	67.9 (44–92)
Warm Season Minimum Temperature [°F] (mean (range))	60.1 (37–81)
Frequency of T_MAX_ ≥ 90 °F (n)	201
Frequency of HI_MAX_ ≥ 86 °F (n)	165
Frequency of T_MAX_ ≥ 85 °F (n)	502
At-Home Deaths (*n*)	6102
Male (*n* (%))	3197 (52.4)
Race, Non-Caucasian (*n* (%))	2276 (37.3)
≥65 years old (*n* (%))	3865 (63.3)
Outside of the Home Deaths (*n*)	34,404
Male (*n* (%))	20,744 (60.3)
Race, Non-Caucasian (*n* (%))	4322 (12.6)
≥65 years old (*n* (%))	19,732 (57.4)
Assessed value of residential building/area ($)(mean (SD))	5910 (28,000)
Assessed value of residential land/area ($)(mean (SD))	408 (463)
Energy efficiency score of residential buildings (mean (SD))	6.25 (0.352)
Year residential buildings were built/renovated (mean (SD))	1970 (15.9)
Decade residential buildings were built/renovated (mean (SD))	1980 (33.9)
Albedo (mean (SD))	0.124 (0.0114)
Impervious Surface Fraction (mean (SD)) [%]	0.787 (0.141)
Number of street trees/CT area (mean (SD))	0.00017 (0.00008)
Proportion of population with at least one disability (mean (SD)) [%]	11.4 (7.4)
Proportion of population that is ≤5 years old (mean (SD)) [%]	16.6 (10.4)
Proportion of population with at least 1 medical illness (mean (SD)) [%]	38.6 (3.19)
Proportion of population that is ≥65 years old (mean (SD)) [%]	10.5 (6.79)
Proportion of population that receives low-to-no income (mean (SD)) [%]	28.0 (17.1)
Proportion of population with limited English proficiency (mean (SD)) [%]	38.5 (17.8)
Proportion of population that is not Caucasian (mean (SD)) [%]	51.6 (30.3)
Proportion of population with utilities excluded from rent (mean (SD)) [%]	82.2 (17.6)
Proportion of population that is unemployed	0.0905 (0.0662)
Gross Rent ($/month)	1180 (471)
Ratio of Females-to-Males	1.06 (0.324)
Gini Coeffcient	0.416 (0.0829)
Population Density (mean (SD)) [people/mile^2^]	23,800 (18,300)

**Table 2.** The relative odds ratio (OR) of dying at home during extreme heat, during the warm season for those who had the following characteristic (Personal, Primary Cause of Death) or lived in a census tract with the characteristic (Social, Environmental), compared with those who did not have that characteristic, Boston, 2000–2015. Individuals were considered to either have a personal modifier or not, or were dichotomized based on being either below the 25th percentile or above the 75th75th percentile of the values for neighborhood modifiers across Boston or dying from that specified primary cause of death. Bold indicates the OR is significant at an *p* < 0.05.

Personal	T_MAX_ ≥ 90 °F		HI_MAX_ ≥ 86 °F		T_MAX_ ≥ 85 °F	

	OR	95% CI	OR	95% CI	OR	95% CI
Female	1.12	(0.94, 1.34)	1.01	(0.83, 1.22)	1.05	(0.93, 1.19)
Not Married	1.14	(0.94, 1.39)	1.13	(0.91, 1.40)	1.08	(0.95, 1.24)
Race, Non-Caucasian	0.90	(0.75, 1.08)	0.99	(0.81, 1.21)	0.99	(0.87, 1.12)
Age < 57	1.02	(0.83, 1.25)	1.13	(0.91, 1.41)	1.03	(0.89, 1.19)
Age > 81	0.88	(0.72, 1.08)	1.06	(0.85, 1.32)	0.94	(0.82, 1.08)
**Social**						
Low Income	**1.30**	**(1.06, 1.59)**	0.92	(0.73, 1.16)	1.14	(0.99, 1.32)
Unemployment	1.03	(0.84, 1.25)	1.07	(0.86, 1.32)	0.98	(0.85, 1.12)
GINI Index	1.00	(0.81, 1.22)	1.01	(0.81, 1.27)	0.98	(0.86, 1.13)
Population Density	1.14	(0.93, 1.39)	1.08	(0.86, 1.35)	1.10	(0.95, 1.27)
Sex Ratio F:M	1.02	(0.84, 1.25)	0.98	(0.79, 1.22)	1.04	(0.91, 1.19)
Utilities not Included	1.13	(0.92, 1.39)	0.89	(0.71, 1.13)	1.08	(0.93, 1.25)
Disability	1.19	(0.97, 1.46)	1.09	(0.87, 1.37)	1.10	(0.95, 1.28)
Children	1.17	(0.95, 1.44)	1.03	(0.82, 1.30)	1.11	(0.96, 1.28)
Elderly	1.01	(0.83, 1.24)	1.01	(0.81, 1.26)	0.99	(0.86, 1.14)
Limited English Prof.	**1.29**	**(1.05, 1.57)**	0.88	(0.69, 1.12)	1.14	(0.99, 1.32)
Race, Non-Caucasian	1.19	(0.97, 1.46)	1.18	(0.94, 1.48)	1.14	(0.98, 1.32)
Medical Illness	0.97	(0.79, 1.20)	0.89	(0.70, 1.12)	1.01	(0.87, 1.17)
**Environmental**						
Energy Effciency	0.96	(0.78, 1.18)	0.95	(0.76, 1.20)	0.94	(0.82, 1.09)
Assessed Value of Res Land/Total Property Area	0.91	(0.74, 1.12)	0.87	(0.69, 1.10)	1.05	(0.91, 1.20)
Assessed Value of Res Building/Gross Floor Area	**1.26**	**(1.04, 1.53)**	1.04	(0.83, 1.29)	1.10	(0.96, 1.27)
Year Built/Renovated	1.13	(0.91, 1.38)	**0.77**	**(0.62, 0.95)**	1.12	(0.97, 1.29)
Trees/CT Area	0.92	(0.75, 1.13)	**0.76**	**(0.60, 0.97)**	0.96	(0.83, 1.10)
Albedo	0.93	(0.78, 1.11)	0.93	(0.76, 1.13)	0.93	(0.82, 1.05)
Impervious Surface Fraction	0.96	(0.78, 1.17)	0.96	(0.77, 1.20)	1.05	(0.91, 1.20)
**Primary Cause of Death**						
Infection	1.46	(0.72, 2.96)	1.72	(0.81, 3.65)	**1.69**	**(1.02, 2.80)**
Cancer	0.84	(0.69, 1.01)	0.85	(0.69, 1.04)	0.93	(0.82, 1.06)
Inflammatory Disease	0.87	(0.35, 2.18)	1.11	(0.44, 2.79)	0.66	(0.33, 1.30)
Endocrine/Nutritional/Metabolic Disease	1.05	(0.71, 1.54)	1.12	(0.75, 1.69)	0.94	(0.72, 1.24)
Mental/Behavioral/Neurodevelopmental Disease	1.26	(0.86, 1.85)	0.76	(0.46, 1.26)	1.02	(0.76, 1.36)
Nervous System Disease	1.24	(0.76, 2.04)	1.06	(0.59, 1.88)	1.07	(0.74, 1.54)
Heart Disease	**1.21**	**(1.01, 1.46)**	1.11	(0.9, 1.37)	1.11	(0.97, 1.26)
Respiratory Disease	1.12	(0.76, 1.67)	1.12	(0.73, 1.74)	1.08	(0.82, 1.43)
Injury/Accident/Event	0.75	(0.49, 1.16)	0.96	(0.62, 1.49)	0.97	(0.74, 1.29)
Self-Harm	0.85	(0.39, 1.86)	0.71	(0.29, 1.78)	0.72	(0.41, 1.24)
Assault-Related Altercation	0.79	(0.4, 1.56)	1.47	(0.82, 2.63)	1.01	(0.66, 1.54)

**Table 3.** The relative odds ratio (OR) of dying outside of the home during extreme heat, during the warm season for those who had the following characteristic (Personal, Primary Cause of Death) or who lived in a census tract with the characteristic (Social, Environmental), compared with those who did not have that characteristic, Boston, 2000–2015. Individuals were considered to either have a personal modifier or be dying from that specified primary cause of death, or not. Bold indicates that the OR is significant at *p* < 0.05.

Personal	T_MAX_ ≥ 90 °F		HI_MAX_ ≥ 86 °F		T_MAX_ ≥ 85 °F	

	OR	95% CI	OR	95% CI	OR	95% CI
Sex	1.03	(0.95, 1.12)	0.97	(0.88, 1.05)	1.03	(0.98, 1.09)
Not Married	1.04	(0.96, 1.12)	1.05	(0.96, 1.14)	1.02	(0.97, 1.07)
Race, Non-Caucasian	0.94	(0.84, 1.06)	1.03	(0.90, 1.17)	0.92	(0.85, 1.00)
Age > 82	0.96	(0.88, 1.04)	0.99	(0.90, 1.09)	**0.93**	**(0.87, 0.98)**
Age < 52	1.07	(0.98, 1.17)	1.03	(0.93, 1.14)	**1.09**	**(1.02, 1.16)**
**Primary Cause of Death**						
Infection	0.81	(0.55, 1.21)	1.11	(0.76, 1.62)	0.99	(0.77, 1.28)
Cancer	0.99	(0.89, 1.10)	1.01	(0.90, 1.14)	1.03	(0.96, 1.11)
Blood/Immune	1.06	(0.62, 1.81)	0.95	(0.51, 1.75)	0.87	(0.59, 1.29)
Endocrine/Nutritional/Metabolic	1.12	(0.94, 1.34)	1.09	(0.90, 1.33)	1.12	(0.99, 1.26)
Mental/Behavioral/Neurodevelopmental	0.84	(0.63, 1.14)	1.01	(0.74, 1.37)	0.9	(0.73, 1.10)
Nervous System Disease	0.96	(0.70, 1.33)	0.83	(0.57, 1.22)	0.88	(0.70, 1.11)
Circulatory Disease	0.99	(0.91, 1.07)	0.92	(0.84, 1.01)	**0.94**	**(0.89, 0.99)**
Respiratory Disease	0.86	(0.71, 1.04)	1.04	(0.85, 1.28)	0.92	(0.80, 1.05)
Congenital Disease	1.54	(0.70, 3.41)	0.24	(0.03, 1.74)	1.4	(0.77, 2.53)
Injury/Accident/Event	1.12	(0.96, 1.31)	1.02	(0.86, 1.22)	1.09	(0.98, 1.22)
Self-Harm	1.03	(0.79, 1.34)	0.87	(0.63, 1.19)	1.12	(0.94, 1.34)
Assault-Related Altercation	1.05	(0.85, 1.30)	1.23	(0.98, 1.53)	1.09	(0.94, 1.26)

**Table 4.** The relative odds ratio (OR) of Boston residents dying outside of the home during extreme heat, during the warm season, for those who had the following characteristic (Personal, Primary Cause of Death) or who lived in a census tract with the characteristic (Social, Environmental), compared with those who did not have that characteristic, Boston, 2000–2015. Individuals were considered to either have a personal modifier or not, or were dichotomized based on being either below the 25th percentile or above the 75th percentile of the values for neighborhood modifiers across Boston or dying from that specified primary cause of death. Bold indicates that the OR is significant at *p* < 0.05.

Personal	T_MAX_ ≥ 90 °F		HI_MAX_ ≥ 86 °F		T_MAX_ ≥ 85 °F	

	OR	95% CI	OR	95% CI	OR	95% CI
Sex	0.81	(0.54, 1.21)	0.74	(0.47, 1.16)	0.77	(0.59, 1.00)
Not Married	1.20	(0.78, 1.83)	1.38	(0.85, 2.24)	1.12	(0.85, 1.47)
Race, Non-Caucasian	1.05	(0.72, 1.54)	1.2	(0.78, 1.84)	1.18	(0.92, 1.52)
Age > 82	0.98	(0.69, 1.39)	1.07	(0.73, 1.56)	0.81	(0.64, 1.02)
Age < 58	1.15	(0.82, 1.60)	**1.53**	**(1.07, 2.18)**	**1.31**	**(1.05, 1.62)**
**Social**						
Low Income	0.75	(0.52, 1.07)	0.81	(0.55, 1.19)	0.99	(0.79, 1.24)
Unemployment	1.03	(0.74, 1.45)	1.16	(0.81, 1.67)	1.06	(0.85, 1.32)
GINI Index	1.34	(0.96, 1.88)	0.91	(0.61, 1.35)	0.92	(0.73, 1.16)
Population Density	0.94	(0.62, 1.43)	0.9	(0.56, 1.43)	0.8	(0.61, 1.06)
Sex Ratio F:M	0.93	(0.66, 1.30)	1.01	(0.70, 1.46)	0.85	(0.68, 1.06)
Utilities not Included	1.07	(0.76, 1.51)	0.67	(0.44, 1.02)	1.09	(0.87, 1.36)
Children	0.81	(0.57, 1.15)	0.96	(0.67, 1.39)	1.07	(0.87, 1.33)
Elderly	**1.49**	**(1.07, 2.07)**	0.92	(0.62, 1.37)	**1.33**	**(1.06, 1.66)**
Limited English Prof.	0.8	(0.56, 1.13)	0.92	(0.63, 1.35)	0.99	(0.79, 1.24)
Race, Non-Caucasian	0.97	(0.70, 1.36)	0.91	(0.62, 1.31)	1.16	(0.94, 1.43)
Medical Illness	1.36	(0.97, 1.91)	1.24	(0.85, 1.80)	1.14	(0.91, 1.43)
Low Income	0.75	(0.52, 1.07)	0.81	(0.55, 1.19)	0.99	(0.79, 1.24)
**Environmental**						
Energy Efficiency	**1.44**	**(1.03, 2.02)**	1.08	(0.73, 1.59)	1.11	(0.88, 1.40)
Assessed Value of Res Land/Total Property Area	0.86	(0.56, 1.32)	0.97	(0.62, 1.52)	0.78	(0.59, 1.03)
Assessed Value of Res Building/Gross Floor Area	0.74	(0.51, 1.07)	0.85	(0.57, 1.27)	0.98	(0.78, 1.23)
Year Built/Renovated	0.83	(0.54, 1.28)	0.85	(0.53, 1.38)	0.81	(0.61, 1.07)
Trees/CT Area	0.86	(0.56, 1.34)	1.12	(0.72, 1.76)	0.95	(0.72, 1.25)
Albedo	1.15	(0.85, 1.56)	0.90	(0.64, 1.25)	1.1	(0.90, 1.34)
Impervious Surface Fraction	0.76	(0.53, 1.10)	0.90	(0.61, 1.33)	0.83	(0.66, 1.05)
**Primary Cause of Death**						
Infection	0.54	(0.07, 4.02)	1.40	(0.60, 1.14)	**0.77**	**(0.62, 0.96)**
Liver Disease	0.46	(0.06, 3.45)	3.58	(0.59, 2.39)	1.14	(0.70, 1.86)
Cancer	0.88	(0.59, 1.31)	0.85	(0.77, 1.48)	0.96	(0.77, 1.21)
Diabetes	1.16	(0.55, 2.44)	1.67	(0.30, 1.93)	1.26	(0.75, 2.10)
Heart Disease	0.9	(0.64, 1.24)	0.65	(0.12, 2.10)	1.26	(0.68, 2.35)
Nervous System Disease	1.11	(0.49, 2.50)	0.78	(0.46, 3.91)	0.8	(0.33, 1.93)
Substance Abuse	**2.88**	**(1.42, 5.86)**	1.16	(0.60, 3.98)	1.75	(0.90, 3.39)
Inflammatory Disease	0.88	(0.21, 3.77)	1.07	(0.69, 8.62)	1.6	(0.56, 4.58)
Cerebrovascular Disease	0.27	(0.04, 1.95)	0.33	(0.06, 3.48)	1.14	(0.42, 3.08)
Digestive System Related Disease	0.66	(0.09, 4.97)	**1.74**	**(1.17, 6.73)**	1.75	(0.89, 3.47)
Unknown Causes	**2.38**	**(1.13, 5.02)**	1.98	(0.82, 4.76)	1.66	(0.92, 2,99)
Assault-Related Altercation	1.49	(0.87, 2.54)	0.92	(0.39, 38.5)	**1.79**	**(1.24, 2.58)**
Injury/Accident/Even	0.8	(0.42, 1.49)	1.42	(0.75, 2.68)	1.11	(0.77, 1.60)
Self-Harm	1.00	(0.36, 2.80)	0.92	(0.28, 2.98)	0.99	(0.50, 1.94)
