# Supplementary material for: The Role of Individual and Small-Area Social and Environmental Factors on Heat Vulnerability to Mortality Within and Outside of the Home in Boston, MA
Source: Climate (Basel). Author manuscript; Available in PMC 2022 Apr 1. (PMC8974638; doi:10.3390/cli8020029)
Supplement: Supplemental Infor [file NIHMS1780865-supplement-Supplemental_Infor.pdf]

Supplemental Information

Figure S1. Neighborhoods of Boston, MA.

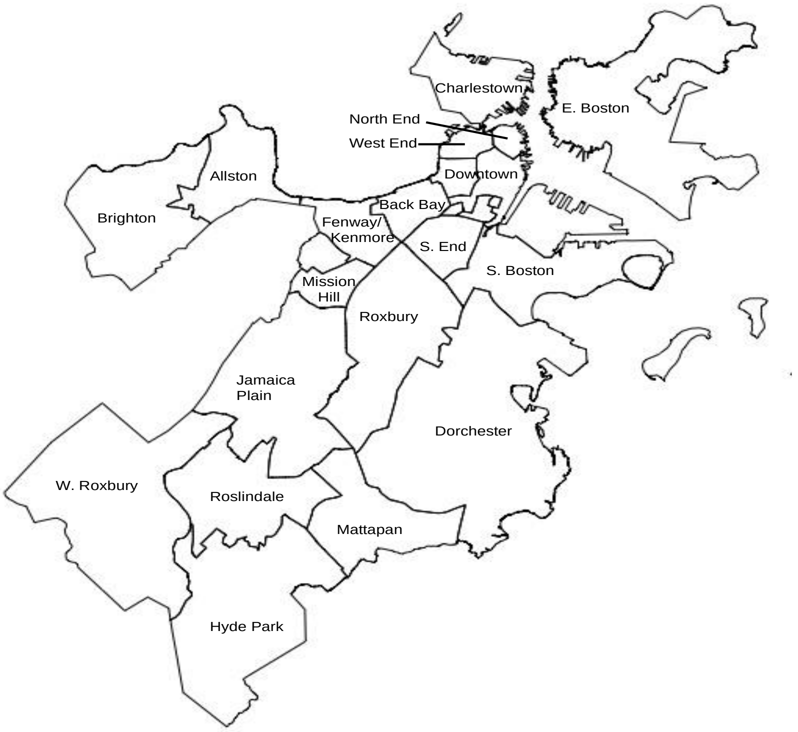

Figure S2. Distribution of the all small-area social and environmental vulnerability factors, Boston, 2000-2015. CT denotes census tract.

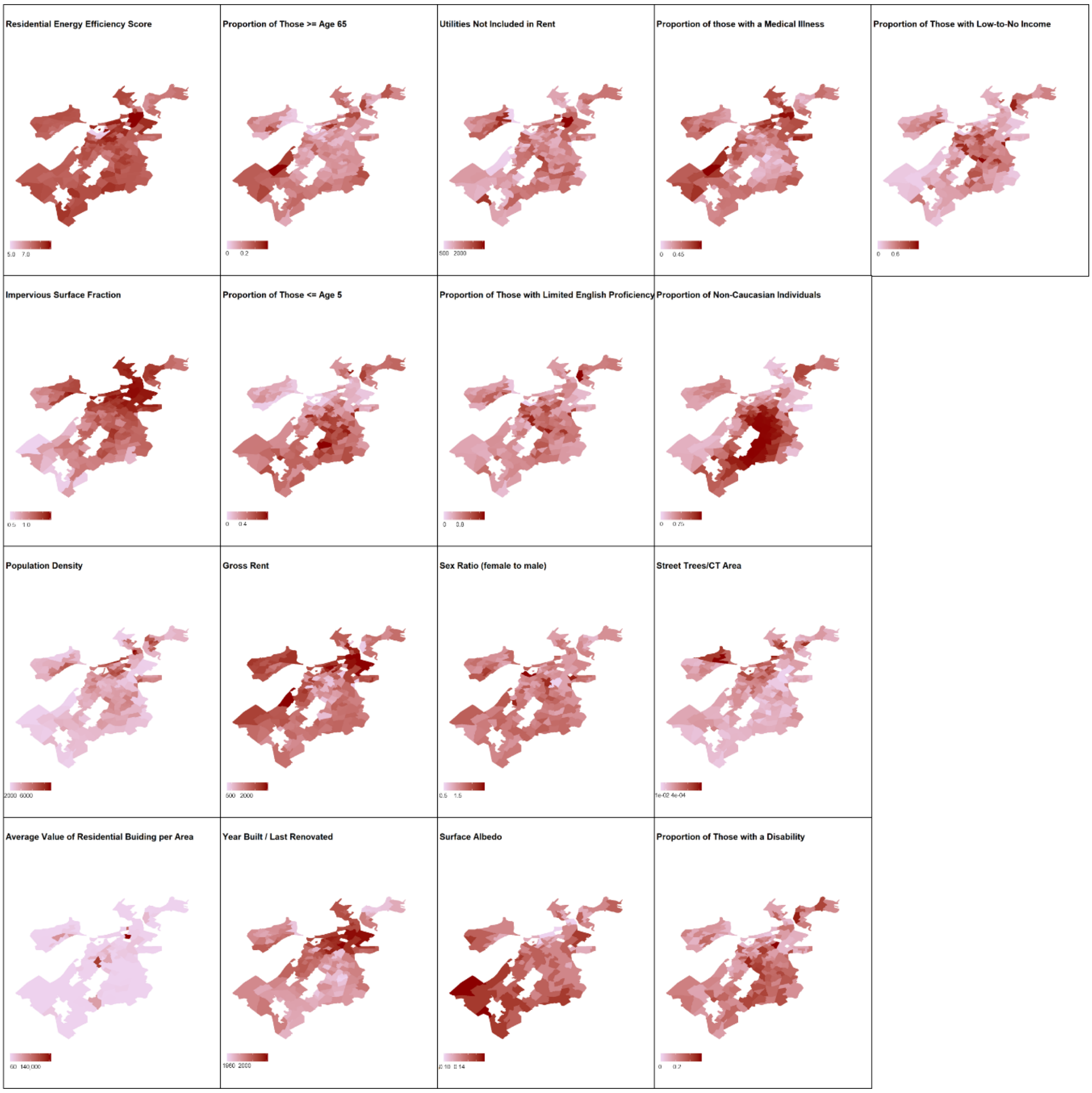

Table S1. Summary of results from GLM analyses limited to only hot days. Bold p-values are significant at an α=0.05 level. All covariates were analyzed using a standardized z-score to center and scale each them respectively.

	Estimate	Standard Error	t value	p-value
Intercept	3.34	0.19	17.28	**<0.001**
TMAX	0.00	0.01	-0.10	0.92
Mean CT Albedo	-0.05	0.04	-1.09	0.28
Trees/CT Area	0.05	0.02	2.09	**0.04**
Mean Value of Building/Area	0.00	0.02	-0.04	0.97
Impervious Surface Fraction	0.07	0.04	1.71	0.09
Population Density	-0.03	0.03	-0.89	0.37
Proportion of those in CT with a Disability	-0.03	0.03	-0.82	0.41
Proportion of those in CT Age≥65	0.08	0.03	3.08	**<0.001**
Proportion of those in CT with Low-to-No Income	-0.04	0.03	-1.17	0.24

Table S2. Summary of results from semiparametric GWR model analyses, restricted to those at-home deaths that occurred on hot days, using the same model that was found to be the best fit from the GLM, but allowing the covariates to vary spatially. All covariates were analyzed using a standardized z-score to center and scale each them respectively. CT indicates census tract and Qu. Indicates quartile.

	Minimum	1st Qu.	Median	3rd Qu.	Maximum	Global
Intercept	-12.88	8.90	20.33	37.44	114.00	15.53
TMAX	-2.68	-0.25	0.01	0.41	1.24	0.13
Mean CT Albedo	-25.64	-8.48	-1.34	6.46	31.79	-0.94
Trees/CT Area	-14.13	-2.27	3.44	6.35	23.58	1.39
Mean Value of Building/Area	-73.78	0.20	2.08	13.28	158.29	-0.31
Impervious Surface Fraction	-20.68	-3.82	1.50	5.99	23.28	2.41
Population Density	-22.83	-4.18	-0.08	9.08	41.24	-0.80
Proportion of those in CT with a Disability	-14.58	-5.37	-3.08	3.19	20.81	-1.31
Proportion of those in CT Age≥65	-14.61	-0.87	2.19	4.28	19.33	2.65
Proportion of those in CT with Low-to-No Income	-46.33	-11.87	-1.27	7.40	15.14	-1.02

Table S3. Summary of results from GLM analyses limited to only days where HI_{MAX}≥86°F at Logan International Airport. Bold p-values are significant at an α=0.05 level. All covariates were analyzed using a standardized z-score to center and scale each them respectively.

	Estimate	Standard Error	t value	p-value
Intercept	3.13	0.11	27.32	**<0.001**
T_{MAX}	0.01	0.00	1.40	0.16
Mean CT Albedo	-0.05	0.04	-1.26	0.21
Trees/CT Area	0.03	0.02	1.35	0.18
Impervious Surface Fraction	0.08	0.04	2.07	**0.04**
Proportion of those in CT with a Disability	-0.04	0.03	-1.30	0.19
Proportion of those in CT Age≥65	0.10	0.02	4.16	**<0.001**
Proportion of those in CT with Low-to-No Income	-0.04	0.03	-1.26	0.21

Table S4. Summary of results from semiparametric GWR model analyses, restricted to those at-home deaths that occurred on days where HI_{MAX}≥86°F at Logan International Airport, using the same model that was found to be the best fit from the GLM, but allowing the covariates to vary spatially. All covariates were analyzed using a standardized z-score to center and scale each them respectively. CT indicates census tract and Qu. Indicates quartile

	Minimum	1st Qu.	Median	3rd Qu.	Maximum	Global
Intercept	-33.11	8.89	16.10	22.86	115.81	14.30
HI_{MAX}	-0.19	0.00	0.08	0.19	1.53	0.14
Mean CT Albedo	-31.80	-7.81	-1.70	3.64	13.02	-1.39
Trees/CT Area	-18.71	-2.80	0.70	7.24	22.70	0.86
Impervious Surface Fraction	-77.55	-5.70	0.32	4.27	23.06	2.37
Proportion of those in CT with a Disability	-35.30	-6.66	-2.13	1.98	17.72	-1.23
Proportion of those in CT Age≥65	-11.19	1.36	4.65	7.41	17.80	3.04
Proportion of those in CT with Low-to-No Income	-22.83	-4.23	-0.67	2.56	134.64	-1.11
Proportion of those in CT who are non-Caucasian	-134.31	-3.14	4.29	8.13	31.32	0.74

Figure S3. Image of triple-decker homes that are common in Boston, MA.

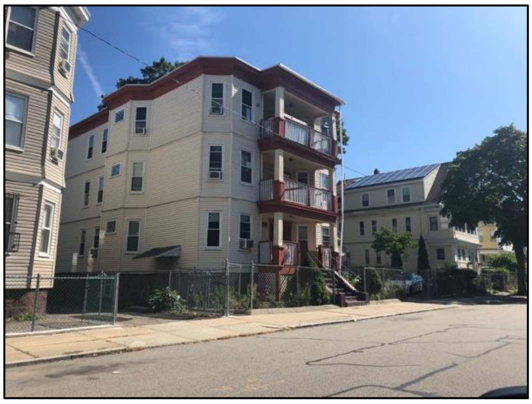

Figure S4. Images of street trees of varying quality, found in (a) Jamaica Plain, (b) Roxbury, and (c) Dorchester, demonstrating examples of the variety in design and quality of street trees.

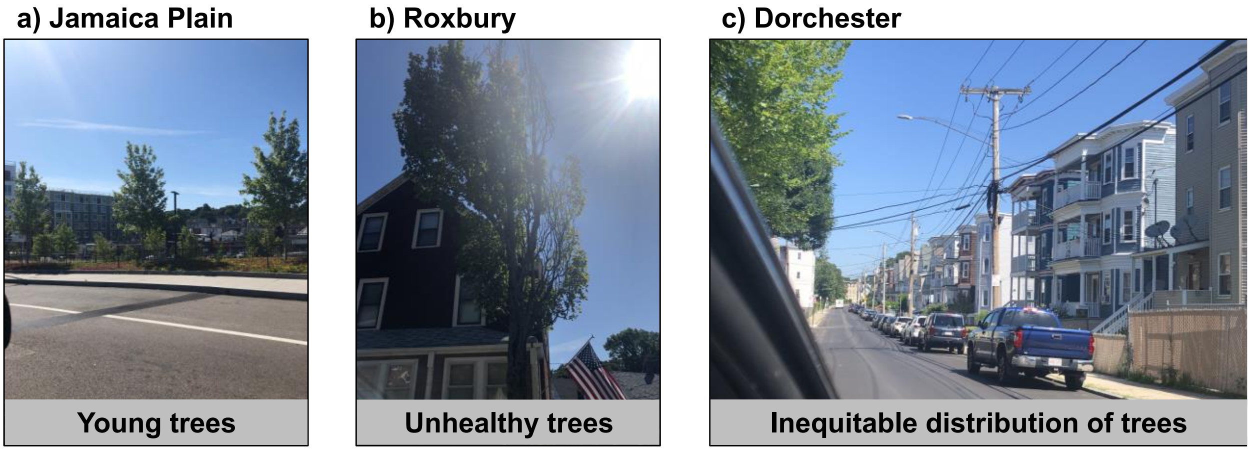

© 2019 by the authors. Submitted for possible open access publication under the terms and conditions of the Creative Commons Attribution (CC BY) license (http://creativecommons.org/licenses/by/4.0/).
